# Impaired Cargo Clearance in the Retinal Pigment Epithelium (RPE) Underlies Irreversible Blinding Diseases

**DOI:** 10.3390/cells7020016

**Published:** 2018-02-23

**Authors:** Eloise Keeling, Andrew J. Lotery, David A. Tumbarello, J. Arjuna Ratnayaka

**Affiliations:** 1 Clinical and Experimental Sciences, Faculty of Medicine, University of Southampton, MP806, Tremona Road, Southampton SO16 6YD, UK; E.Keeling@soton.acuk (E.K.); A.J.Lotery@soton.ac.uk (A.J.L.); 2 Eye Unit, University Hospital Southampton NHS Foundation Trust, Southampton SO16 6YD, UK; 3 Biological Sciences, Faculty of Natural & Environmental Sciences, Life Science Building 85, University of Southampton, Highfield Campus, Southampton SO17 1BJ, UK; D.A.Tumbarello@soton.ac.uk

**Keywords:** Retinal Pigment Epithelium (RPE), endosomes, phagosomes, lysosomes, autophagy, RPE cultures, Age-related Macular Degeneration (AMD)

## Abstract

Chronic degeneration of the Retinal Pigment Epithelium (RPE) is a precursor to pathological changes in the outer retina. The RPE monolayer, which lies beneath the neuroretina, daily internalises and digests large volumes of spent photoreceptor outer segments. Impaired cargo handling and processing in the endocytic/phagosome and autophagy pathways lead to the accumulation of lipofuscin and pyridinium bis-retinoid A2E aggregates and chemically modified compounds such as malondialdehyde and 4-hydroxynonenal within RPE. These contribute to increased proteolytic and oxidative stress, resulting in irreversible damage to post-mitotic RPE cells and development of blinding conditions such as age-related macular degeneration, Stargardt disease and choroideremia. Here, we review how impaired cargo handling in the RPE results in their dysfunction, discuss new findings from our laboratory and consider how newly discovered roles for lysosomes and the autophagy pathway could provide insights into retinopathies. Studies of these dynamic, molecular events have also been spurred on by recent advances in optics and imaging technology. Mechanisms underpinning lysosomal impairment in other degenerative conditions including storage disorders, α-synuclein pathologies and Alzheimer’s disease are also discussed. Collectively, these findings help transcend conventional understanding of these intracellular compartments as simple waste disposal bags to bring about a paradigm shift in the way lysosomes are perceived.

## 1. Introduction

**Figure 1:**
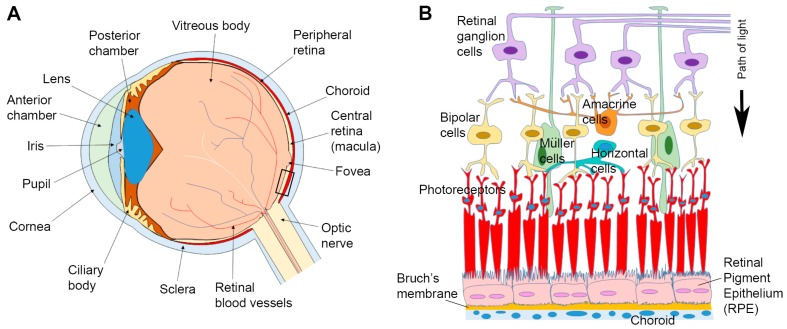
Anatomy of the eye and arrangement of cells in the retina and associated tissues. (**A**) Schematic diagram of the eye in cross-section. (**B**) Enlargement of the area indicated in box (**A**) showing relative position of the Retinal Pigment Epithelium (RPE) in relation to other tissues. Sandwiched between the overlying neuroretina and the underlying Bruch’s membrane/choroid, the RPE monolayer marks the important blood-retinal-barrier. A black arrow indicates the pathway of light.

The Retinal Pigment Epithelium (RPE) is a monolayer of cells which lies beneath the neuroretina. Amongst its many functions, the RPE internalises photoreceptor outer segments (POS) from overlying photoreceptors as part of the daily visual cycle. Each RPE cell may ‘serve’ up to 45 rod or cone photoreceptors where a daily renewal process results in ~10% of their volume being shed and subsequently phagocytosed by adjacent RPE [1,2]. Microvilli on the apical RPE surface interdigitate and surround these photoreceptor tips in order to enhance phagocytosis [3,4,5]. On the basolateral side of RPE cells, highly convoluted basal infolds increase the surface area for optimal absorption of oxygen and nutrients. The substantial metabolic waste generated in the outer retina is removed via the underlying choriocapillaris [6,7]. Sandwiched between this dense capillary network and the RPE is a porous tissue of 2–4 μm thickness referred to as Bruch’s membrane (BrM) which supports the overlying cell monolayer (Figure 1). The daily internalisation of POS makes the RPE one of the most phagocytic cells in the body. This, coupled with the fact that in-situ, RPE are largely post-mitotic, makes the proteolytic burden in these cells considerable. Studies in Rhesus monkeys showed that each cell is exposed to 2000 discs per day in the parafovea, 3500 in the perifovea and approximately 4000 in the periphery, with each RPE processing up to a billion photoreceptor disks over a 70-year period [2]. The high photo-oxidative retinal environment imposes further stresses amongst which the modification of intracellular cargos within membrane-bound vesicles impairs their clearance and turnover in senescent RPE. Consequently, cargo handling in the RPE endocytic and autophagy pathways has garnered considerable attention, as its dysregulation is associated with several retinopathies [8,9,10,11,12,13,14]. Given recent discoveries revealing new roles for lysosomes and autophagosomes, and the ability to study their dynamic behaviour using novel imaging technologies, we review the topic of how RPE cells cope with the high proteolytic burden in the senescent retina, and why its impairment leads to irreversible blindness. We also consider how lysosomal impairment in other degenerative conditions could provide insights into shared pathogenic processes associated with cargo handling in the mammalian cell.

## 2. Components of the Endocytic/Phagosome and Autophagy Pathways

### 2.1. The Endo-Lysosomal Pathway

The function of the endocytic pathway is to traffic and sort cargoes originating from the extracellular environment and the plasma membrane. This cargo-handling pathway may be considered analogous to an elaborate pipeline with various intermediary junctions for the entry/exit of molecules with a common convergence point at lysosomes. During endocytosis, cargo internalisation may initiate at clathrin-coated pits on the plasma membrane that bud into vesicles. Early endosomes (EEs) are relatively small and range between 200–500 nm with tubular/vacuolar domains and are found in the peripheral cytoplasm in close proximity to the plasma membrane [15]. Individual EEs move along microtubules in a saltatory manner [16]. EEs are considered to be the major sorting point in the endocytic pathway, receiving cargos via the clathrin-mediated pathway as well as other routes including ARF6-dependent and caveolar pathways [17]. Hence, EEs internalise the plasma membrane as well as extracellular materials. It has been estimated that in a typical mammalian cell, 50–180% of the plasma membrane surface area is recycled each hour [18]. The Rab family of small GTPases act as molecular switches that alternate between the activated GTP-bound and the inactivated GDP-bound forms. These proteins have different corresponding host organelles, and are hence regarded as markers of distinct compartments. Members of the Rab family mediate vesicle maturation through interactions with various effector proteins [19]. For instance, EEs contain Rab-4, Rab-5 and Rab-11, which provides a means for sorting to target destinations [20]. A noteworthy component recruited to the cytosolic surface of EEs as well as maturing late endosomes (LEs) is the ‘retromer’; a multimeric complex composed of sorting nexins and associated proteins that mediates the retrograde retrieval of cargos from endosomes to the plasma membrane or to the trans-Golgi network for re-use [21]. Another complex is the large, multimeric proton pump termed vacuolar ATPase (vATPase), which functions as a transmembrane conduit for the acidification of endosomes as well as lysosomes [22]. Consequently, the luminal environment within EEs is weakly acidic (pH 5.9–6.8). EEs are sites at which intraluminal vesicles (ILV) form; a process that occurs involving clathrin and components of the endosomal sorting complex required for transport (ESCRT) which sorts ubiquitinated membrane proteins into ILVs to form multivesicular bodies (MVBs). MVBs may therefore contain several ILVs [23,24]. These MVBs and a proportion of EEs subsequently mature and become LEs.

LEs are derived from vacuolar domains of EEs, which also contain ILVs as well as other incoming particles such as viruses. EEs that are positive for Rab-5 recruit Rab-7, which results in Rab-5/Rab-7 hybrid endosomes. Such hybrid vesicles, however, are short-lived, as Rab-5 appears to be lost within minutes to be rapidly replaced by Rab-7 [25]. This process of LE maturation is referred to as ‘Rab conversion’ [26]. An alternative mechanism of LE formation proposes a fission event with separate portions of the hybrid vesicle containing Rab-7 that serves to transport cargos to stable LE compartments prior to delivery to lysosomes [27]. These alternative models of LE formation may not necessarily be mutually exclusive, as separate mechanisms may co-exist depending on their spatial subcellular localisation or the type of cargo. As endosomes mature, their saltatory movement in the cell periphery change to rapid long-range oscillations with net displacement towards the perinuclear zone, where lysosomes reside [16,28]. The formation of LEs is followed by an elaborate process of maturation, the details of which are reviewed elsewhere [29,30]. This transformation entails a complete makeover of LE, the end-product of which bears little resemblance to precursor EEs. For instance, tubular extensions of EEs are lost as endosomes become increasingly vesicular shaped. Mature LEs also have a larger diameter (250 nm^–1^ μm) compared to EEs, whilst increasing acidification results in luminal values between pH 4.9–6.0. Decreasing pH levels are important for the activity of luminal hydrolytic enzymes and for acquiring an identity characteristic of LEs [31]. Other changes associated with LE maturation includes the Rab GTPase switch, conversion to distinct phosphoinositide species, association with Arf1/COP1, ILV biogenesis and acidification [29]. The long-range movement of LEs primarily occurs along microtubules and is dependent on molecular motors such as kinesin and dynein. These molecular engines, along with specific multi-subunit tethering complexes, SNARE proteins, and actin-dependent myosin motors, are involved in the fusion of endosomes with each other [32,33]. This molecular ‘refining’ of LEs may not only be considered as a means by which late compartments are distinguished from Rab-4, 8, 10, 11, 13, and 22a positive recycling EEs that return to the plasma membrane [34], but also as a means to funnel specific cargoes destined for degradation to lysosomes. A similar process of maturation may occur in autophagosomes [35] and phagosomes [36], prior to fusion with LEs and lysosomes. 

**Figure 2:**
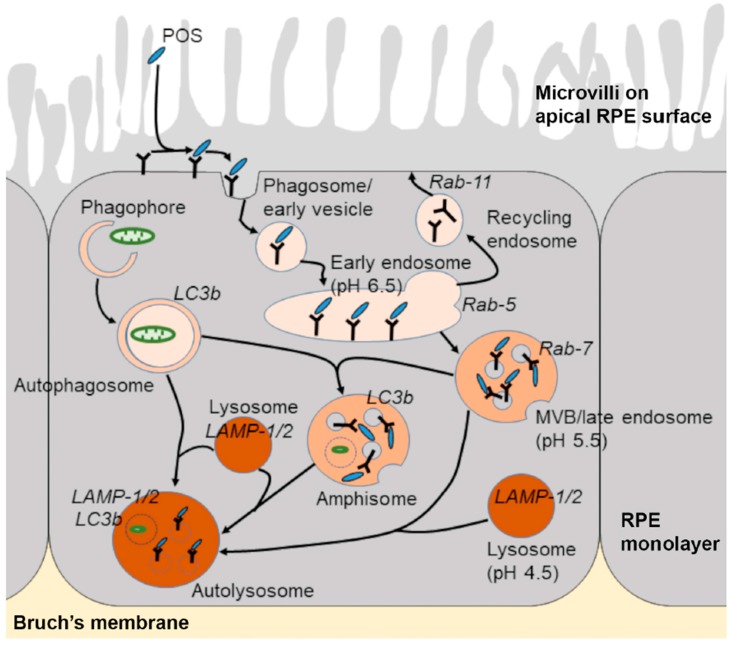
Schematic diagram showing the endo-lysosomal and autophagy pathways. Shed photoreceptor outer segment (POS) disks bind to the apical RPE surface following light onset and are internalised via a series of phagocytic and endosomal compartments prior to converging with lysosomes for degradation. Vesicles involved in cargo recycling as well as components of the autophagy pathway are also shown. Post-mitotic RPE cells are required to rapidly engulf and digest high volumes of POS daily throughout life, which results in the accumulation of partially-degraded and chemically-modified cargos within mature compartments in later life and the development of several binding diseases for which there are no effective treatments.

The biogenesis of lysosomes is thought to occur via EEs, although the full details of this process remains to be elucidated. Lysosomal constituents such as newly synthesized acid hydrolases are trafficked from the trans-Golgi network via mannose 6 phosphate receptors (M6PRs) to endosomes. Acid hydrolases thus initially reside in endosomes and may be subject to further modification prior to becoming active enzymes within lysosomes [37]. Although M6PR pathway is an important route of delivering soluble luminal proteins to lysosomes, it is by no means exclusive, as M6PR-independent mechanisms have also been described [38]. The limiting outer membrane of lysosomes consist of a single phospholipid bilayer on which lysosome-associated membrane protein (LAMP-1) and LAMP-2 constitute a majority of membrane proteins [39]. Hence, LAMP proteins are typically used as markers of lysosomes. The limiting membrane is protected from auto-digestion by resident lytic enzymes through glycosylation of lysosomal integral membrane proteins LAMPs and CD36. These organelles form part of a family of communicating, acidic, vesicular compartments (Figure 2), where intra-vesicular pH ranges from 3.8 to 5.0 [40], and their diameters can vary between 200 nm and 1 μm [41,42]. To facilitate cargo degradation, lysosomes contain over 50 lysosomal membrane proteins, including an array of channels/transporters such as vATPase, as well as 60 different types of hydrolytic enzymes [39,43]. This luminal environment provides ideal conditions for the activity of lysosomal enzymes, which according to their preference for substrates are grouped as lipases, glycosidases, acid phosphatases, proteases, sulfatases and nucleases. Internalised cargoes are broken down to generate monosaccharides, amino acids and free fatty acids amongst other compounds [43,44]. The products of lysosomal digestion are eventually transported to the cytoplasm for use in a variety of biosynthetic activities. Lysosomes are typically found in the perinuclear region and are transported along microtubules by the aforementioned kinesin and dynein motors [42]. Lysosomes also switch between kinesin-mediated plus-end and dynein-mediated minus-end movement; hence, they appear to frequently alter direction in live-cell microscopy [45,46]. Vesicular associations with kinesin were shown to occur at sites where late compartments make contact with the endoplasmic reticulum (ER). Here, kinesin-1 is transferred from protrudin, an ER protein, to the motor adaptor FYCO1 on LEs. Repeated LE-ER contacts promoted microtubule-dependent LE movement to the cell periphery and fusion with the plasma membrane inducing neurite outgrowth [47]. Impairment of vesicle contact with the ER is associated with disorders including Niemann-Pick disease (NPD), as metabolic factors such as cholesterol levels were demonstrated to regulate the intracellular positioning of LEs [48]. These findings reveal the importance of LE and lysosome interactions with cytoskeletal elements via motor proteins and potential consequences associated with their disruption. However, under normal physiological conditions, the population of lysosomes appear to be largely stable over time, and a single cell may contain up to several hundred lysosomes at any given time [49,50]. 

### 2.2. The Phagocytic Pathway

Another method of cargo internalisation is via the formation of phagosomes. POS for instance is primarily internalised via phagosomes within RPE cells, which appear as 1 μm diameter inclusions [51,52]. The process of phagosome formation/maturation has been well-characterised, particularly in cells involved with immune signalling and pathogen elimination [53], and occurs in a series of sequential steps. The polymerisation of actin molecules under the cell surface initiates plasma membrane protrusions that bring targets in contact with the phagocytic surface [54]. Receptors such as receptor tyrosine kinase c-mer (MerTK), MARCO, FcγRs and TIM4 bind targets to initiate a signalling cascade. Different phagocytes may contain a distinctive repertoire of receptors depending on the cell type and specificity for certain target molecules. Binding to target molecules is typically associated with lateral clustering of receptors [55]. Membrane protrusions then coalesce at distal margins to surround and seal the target molecule within the nascent phagosome [56]. The phagosome then undergoes a series of fusion and fission events with the aforementioned constituents of the endocytic pathway through which it matures from an early phagosome to a late phagosome and eventually to a phagolysosome. Early phagosomes can fuse with EEs. Although the primary aim is to degrade cargoes, certain types of target molecules could also be recycled back to the plasma membrane or directed to the trans-Golgi network [56,57]. In this respect, cargo sorting in early phagosomes appears to be analogous to events in the early endocytic pathway. Recycling phagosomes are reported to be positive for Rab-4, Rab-10 and Rab-11 [58,59]. In common with endosomes, the acquisition of Rab-5 by early phagosomes is an important event allowing vesicle maturation including subsequent recruitment of Rab-7 [60]. This ‘Rab switching’ is necessary to facilitate the maturation of early phagosomes to late phagosomes and is associated with recruitment of additional vATPases, enrichment of ILVs, increased luminal pH as well as the inward migration for fusion with lysosomes [61,62]. In this final stage of forming phagolysosomes, vesicles acquire LAMP-1 and LAMP-2 positivity [63]. Studies in dendritic cells show that phagosome maturation including fusion with lysosomes is influenced by the stromal interaction molecule (STIM1), an ER protein that detects Ca^2+^ levels in the ER. STIM1-dependent Ca^2+^ regulation also promotes the delivery of endo-lysosomal enzymes to phagosomes, revealing the importance of calcium in these processes [64]. The acidification of the phagosome luminal environment may result in pH values as low as ≥5.0 in certain cell types [65].

### 2.3. The Autophagy Pathway

Autophagy is primarily a non-selective process through which cells degrade intracellular constituents as part of a homeostatic response to nutrient and amino acid starvation. Alternatively, it can function as a selective pathway to degrade misfolded or aggregated proteins as well as damaged organelles and act as a quality control mechanism. Therefore, autophagy is a useful mechanism through which cells cope with stress, low energy stores and associated effects of ageing. The term autophagy, meaning ‘self-eating’ has taken centre-stage following the 2016 Nobel Prize award in Physiology/Medicine to Yoshinori Ohsumi for his seminal work characterising components of the autophagy machinery. Three types of autophagic pathways have been described referred to as (1) microautophagy, (2) chaperone-mediated autophagy, and (3) macroautophagy [66]. Microautophagy is a process through which small quantities of cytoplasm non-selectively and directly enters lysosomes for degradation. In chaperone-mediated autophagy, cytosolic proteins such as the amyloid precursor protein (APP) containing the KFERQ motif are preferentially targeted to lysosomes for degradation via interactions with the hsc70 complex [67]. Macroautophagy involves the large-scale degradation of cytoplasmic constituents which are encapsulated by a distinct double membrane-bound organelle referred to as the autophagosome. EEs and LEs also fuse with autophagosomes to create intermediate compartments termed amphisomes. Although amphisomes are observed in many different cell types, whether they have any cellular functions other than acting as a precursor structure remains to be established [68]. Autophagosomes and amphisomes fuse with lysosomes to form autolysosomes (Figure 2), which results in the degradation of cargos and the recycling of cellular components such as amino acids and lipids [66]. One of the primary regulators of the autophagy pathway is the nutrient sensor mechanistic target of rapamycin complex 1 (mTORC1). In response to nutrient deprivation or amino acid starvation, mTORC1 becomes inhibited as a result of the modulation of various upstream regulators, such as AMP-activated protein kinase (AMPK). This subsequently leads to the activation of the autophagy-initiating ULK1 complex, which comprises ULK1-FIP200-ATG13. This complex triggers a cascade of events leading to the activation and recruitment of primary autophagy regulators that facilitate the encapsulation of cargo by a growing autophagosomal membrane. Thus formed, the autophagosome matures, in a similar manner to endosomes along the endo-lysosomal pathway, leading to eventual formation of an autolysosome. Another function of mTORC1 is its ability to regulate the behaviour of the transcription factor EB (TFEB). This transcription factor along with other members of the microphthalmia-transcription factor E subfamily (MiT/TFE) were discovered to regulate genes encoding for lysosomal and autophagy proteins [69,70,71]. mTORC1 phosphorylates TFEB to retain this transcription factor in the cell cytoplasm [72,73]. However, under nutrient-poor conditions, mTORC1 inhibition coupled to lysosomal Ca^2+^ release activates calcineurin (a calcium-dependent phosphatase) to dephosphorylate TFEB, which promotes its translocation to the nucleus to activate a group of promoters referred to as coordinated lysosomal expression and regulation (CLEAR), and induce the expression of lysosomal hydrolases and autophagic proteins [74,75]. These findings shed new light onto previously unknown links between lysosomes and the autophagy pathway as well as regulatory mechanisms governing their behaviour.

## 3. Impaired Cargo Handling and Proteolysis Underlies Several Chronic Degenerative Diseases

Dysfunctional lysosomes accumulate undigested cargo/substances leading to a group of diseases known as lysosomal storage disorders (LSDs) [76]. These are a family of inherited disorders which affect different cell-types, tissues and organs [41]. It appears that two-thirds of LSDs are associated with brain lesions [77]. A majority of LSDs are caused by mutations affecting genes coding for specific lysosomal hydrolases, which leads to abnormal accumulation of macromolecular proteins within lysosomes [78,79]. However, other forms of the disease carry mutations in non-enzymatic proteins [39], adding a further complexity and clinical heterogeneity to LSDs. In most LSDs, autophagic flux is also reduced [80,81], evidenced by elevation of autophagic substrates and the autophagosome-associated marker LC3b [76]. The aforementioned NPD is a group of autosomal recessive diseases typified by defects in lysosomal homeostasis and function. In NPD type-C, cholesterol is mis-trafficked through the endocytic pathway resulting in an accumulation within LEs and lysosomes [82]. This is associated with Alzheimer’s disease-like pathology including formation of neurofibrillary tangles, increased processing of APP as well as endosomal abnormalities [83,84].

Impairment of the autophagy and ubiquitin–proteasome pathways also underlies conditions such as Parkinson’s disease (PD), Alzheimer’s disease (AD) and Huntington’s disease as well as other neurological disorders such as amyotrophic lateral sclerosis. PD for instance is characterised by abnormal accumulation of α-synuclein in the shape of Lewy bodies and Lewy neurites. These neuronal inclusions are observed in the frontal and parietal cortex, para-hippocampal and cingulate gyri, the insula, basal nucleus of Meynert and in the diencephalon [85]. PD is also associated with mutations in lysosomal ATPase as well as parkin which is an essential component for the autophagic clearance of damaged mitochondria, called mitophagy [86]. In addition, mutant α-synuclein fails to be translocated to lysosomes for degradation [87]. This failure of clearance may explain the exceptionally high affinity of α-synuclein for lysosomal membrane receptors, which are required for the autophagic pathway. This interaction is thought to block lysosomal uptake, inhibiting the degradation of mutant α-synuclein as well as other autophagy substrates [76,87]. Cathepsin D has been shown to be a major protease involved in lysosomal clearance of α-synuclein in cellular and animal models of PD. High levels of Cathepsin D have been shown to reduce α-synuclein aggregation and toxicity [88]. However, Cathepsin D activity decreases as a result of insufficient endosomal sorting and protease trafficking to lysosomes, resulting in reduced α-synuclein clearance [89]. Elsewhere, an inability to complete autophagy leads to the accumulation of ubiquitinated and aggregate-prone polypeptides in the cytoplasm, including p62/SQSTM1, α-synuclein as well as the Huntingtin protein in Huntington’s disease [90,91,92]. Although α-synuclein is cleared by autophagy [93], it also contributes to disease by reducing the efficiency of autophagosome formation [94]. AD brains, in contrast, are characterised by aggregation of the APP-derived cytotoxic amyloid beta proteins (Aβ). The endo-lysosomal and autophagy pathways in AD neurons have garnered considerable attention as their impairment is linked with dementia [95,96,97]. Following secretion to the extracellular environment, Aβ accumulates in senile plaques which also contain many lysosomal enzymes including Cathepsin D [98]. Enlarged EEs is one of the earliest known neuropathological features of AD, reported decades before clinical symptoms develop [99]. Aβ has been identified in intra-neuronal sites within the cargo-sorting pathway, in Rab-5 positive endosomes [100], in autophagic vacuoles [101,102] and in MVBs [103] within AD neurons. Dystrophic neurons in AD brains also contain increased numbers of autophagic vacuoles [104], which includes autophagosomes, autolysosomes and lysosomal dense bodies [105], indicating a gross dysfunction of the autophagic pathway. This accumulation of vacuoles is thought to arise from a defect in autophagy vacuole clearance rather than being due to an increase in autophagy induction [98]. The inefficient fusion between constituent vesicular compartments may be the underlying cause as there is evidence of immature autophagic vacuoles and lysosomal dense bodies in the cytoplasm. The accumulation of cathepsin-positive autophagic vacuoles containing LC3 (a membrane associated autophagic protein which is normally degraded rapidly after autolysosome formation), further suggests a defect in protein degradation within autolysosomes [106]. Moreover, the ε4 allele of apolipoprotein E (known to be the strongest genetic risk factor for AD) exacerbates dysregulation of the endosomal pathway [107] and induces leakage of lysosomes [108]. Impaired endosomes/lysosomes can also be observed in congenital disorders such as in Down syndrome [109], demonstrating that problems in cargo trafficking and proteolytic processing can underpin a wide spectrum of neurological conditions. A noteworthy feature of neurons is the presence of ~40 nm diameter sized synaptic vesicles (SVs) within pre-synaptic terminals and axons. SVs also contain molecules such as Rab proteins, ATPase and SNAREs [110], and are acidified and regulated in a manner analogous to larger endocytic compartments. Here, their cargoes consist of neurotransmitters instead of cell-surface receptors and other molecules. Our studies have revealed how SVs are shared between adjacent pre-synaptic terminals [111], signal at extra-synaptic sites [112], and how distinct populations of SVs can be harnessed to modulate synaptic plasticity [113]. The aforementioned findings suggest that their larger counterparts in the endocytic pathway are also regulated with high precision and hence dysregulation of cargo handling may represent some of the earliest stages in congenital as well as chronic degenerative diseases. 

## 4. RPE Impairment and Retinal Disease 

Of all the tissues in the outer retina, the relative positioning of the RPE monolayer places these cells under considerable mechanical and physiological stress (Figure 1B), a process that becomes exacerbated with increasing age. Gradual dysfunction of the RPE is, therefore, considered to be a key driver of disease leading to conditions such as age-related macular degeneration (AMD) [8,9,10], Stargardt disease [11,12,13] and choroideremia [14] in which impairments of the endocytic/autophagy-lysosomal pathways are implicated. Collectively, retinopathies are responsible for a large proportion of chronic degenerative diseases, contributing to diminished quality of life and increased morbidity. AMD for instance is the leading cause of irreversible sight-loss amongst adults in developed societies. Globally, early AMD is estimated to affect ˃150 million individuals, whilst sight-threatening late-stage forms are thought to affect ~10 million individuals [114]. Early disease is typified by the appearance of sub-RPE protein/lipid deposits known as drusen. This asymptomatic phase may progress to intermediate and advanced stages in which two broadly defined phenotypes are recognised: geographic atrophy (GA) and neovascular AMD (nvAMD) [7]. In GA, progressive RPE degeneration results in the death of overlying photoreceptors, whilst nvAMD is characterised by formation of new/leaky vessels which exude fluids to damage the retina. The latter is also associated with breaks in the blood–retinal barrier, subretinal fluid accumulation and formation of scar tissue. AMD is a multifactorial disease driven by a combination of genetic as well as non-genetic/environmental risk factors [7]. Although our work has contributed significantly to identifying the genetic risks of AMD [115,116], the mechanisms driving disease at the level of cells and tissues in the outer retina remain to be fully understood. Impairment of the RPE is regarded to be a major feature of AMD, as multiple disease pathways converge to disrupt this important monolayer [7,117]. Amongst pathogenic events targeting the RPE, damage to lysosomal-mediated pathways, originating from both the endocytic and autophagy routes feature prominently as a trigger/driver of retinopathy, and has been the focus of considerable attention [8,9,10,11,12,13,14,57].

## 5. Impairment of Lysosomes and other Components of Cargo Handling in RPE Cells 

**Figure 3:**
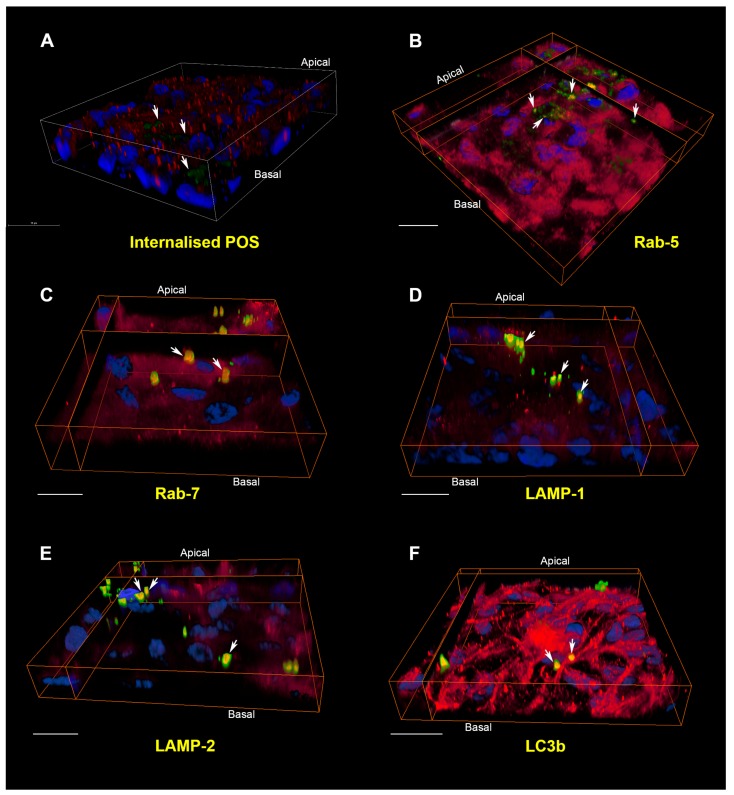
The internalisation pathway of photoreceptor outer segment cargo in RPE cells. (**A**) A pulse-chase assay in cultured RPE monolayers showed bound FITC-POS in green (arrows) at zero hours. Nuclei are stained with DAPI (blue) whilst Rab-5-positive vesicles appear in red. Note the lack of POS co-localising with early compartments at this initial stage. Scale bar in confocal orthogonal cross-section corresponds to 20 μm; (**B**) 4 h following pulse-chase, POS may be observed in Rab-5 positive early compartments and appear as discrete yellow vesicles. (**C**) By 6 h, cargos had been trafficked to Rab-7 labelled late endosomes/phagosomes. (**D**) After 12 h, POS appear in vesicles labelled with the lysosomal marker LAMP-1. (**E**) At 24 h, POS were found in vesicles positive for the mature/late lysosomal marker LAMP-2. Note the perinuclear distribution typical of late compartments. (**F**) POS persisted in vesicles labelled with LC3b, a marker of autophagy bodies, as late as 48 h. The trafficking and processing of POS cargo through distinctive compartments can thus be visualised in cultured ARPE-19 cells. In all experiments, each compartment was labelled with a specific antibody (red), whilst POS-FITC and nuclei appear in green and blue, respectively. Areas of co-localisation between POS and the vesicle-specific marker appear yellow and are denoted by white arrows. POS that are trafficked through other compartments at a given time appear green, whilst areas of red indicate vesicles devoid of any cargoes. Scale bars in (**B**–**F**) correspond to 20 μm. Panel **A** shows a conventional confocal z-stack, whilst panels (**B**–**F**) show three-dimensional RPE monolayers captured using a Leica SP8 confocal microscope (LAS X) and reconstructed using Amira 6.1 software.

To cope with the aforementioned high proteolytic burden, the RPE have a highly active lysosomal system which constitutes a large proportion of the cell’s cytoplasmic volume [118]. There are three well-recognised receptors critical for regulating the internalisation of POS: MerTK, αvβ5 integrin, and the macrophage phagocytosis receptor CD36. These operate in a circadian rhythm where αvβ5 integrin is required for mediating POS binding to RPE [119]. By contrast, CD36 causes the internalisation of POS [6] whilst MerTK activates phagocytosis [120,121]. Upon ingestion, phagosomes carrying POS fuse with lysosomes to form phagolysosomes. Of the several lysosomal enzymes mediating proteolytic breakdown of POS, Cathepsin D appears to be prominent in RPE cells [10]. This process of POS internalisation and cargo degradation can be studied using in-vitro cultures such as those utilised in our laboratory (Figure 3) [122,123]. ARPE-19 grown on transwell inserts for two months allowed cells to form confluent, hexagonal, pigmented monolayers expressing the cell-specific marker RPE65 and displaying apical and basolateral structural features of native RPE. ARPE-19 is a widely utilised cell-line shown to readily internalise isolated POS prepared from bovine or porcine sources [119]. Desirable structural features and functional specialisation were similarly observed in our primary mouse RPE monolayers [123] cultured from postnatal day 10–12 C57BL/6 mice as reported by others [124,125]. Isolated POS were fluorescently labelled with Alexa Fluor FITC 488, which bound to MerTK and αvβ5 integrin receptors on the apical RPE surface [123]. Phagocytic ligands including MFG-E8 have been shown to be important for POS binding via the αvβ5 integrin receptor [52]. These opsonisation ligands may either be secreted apically by RPE or provided through heat-inactivated foetal calf/bovine serum used in cultures [125]. Macular RPE cells, which are particularly prone to damage in conditions such as AMD, have a mean ratio of photoreceptors per RPE cell which is higher than RPE in the peripheral retina [126]. Although this cannot be recapitulated with any accuracy under culture conditions, we followed a well-established protocol of feeding 4 μg/cm^2^ of POS-FITC to cultures as described previously [127]. We also used a pulse-chase assay that involved lowering the culture temperature to 17 °C for 30 min, prior to POS feeding, following which cells were maintained at this lower temperature for a further 17 min to allow maximal POS binding with minimal cargo internalisation. Following this, the POS-FITC-containing medium was aspirated and cultures washed in complete media to remove any unbound molecules [128]. Conditions of our pulse-chase assay also conformed to ideal conditions in which POS binds to the αvβ5 integrin receptor with minimal internalization [129,130]. Cultures were immediately returned to the physiological temperature of 37 °C to initiate the internalisation of cargo.

The molecular events involved in POS recognition and internalisation are better understood compared to those associated with cargo degradation. POS are internalised within RPE phagosomes, which can be observed morphologically in toluidine-blue-stained sections [131], by cryo-immuno electron microscopy [57], fluorescent labelling [52,125] and, more recently, by correlative light-electron microscopy [132]. Nascent phagosomes are enriched with annexin A2, whilst siRNA knock-down of annexin A2 results in impaired POS internalization [133]. POS phagosomes have also been shown to associate with myosin-7a and kinesin-1 as they traffic away from the apical RPE surface. Impaired phagosome localisation and degradation in aged mice lacking kinesin-1 light chain 1 resulted in a phenotype resembling features of AMD [134]. Our studies demonstrated labelling of internalised POS cargoes with Rab-5 and Rab-7 markers for the first time [123]. However, we are unable to comment with any certainty on whether these POS cargoes were trafficked through phagosomes or endosomes. It is likely that both pathways may be used by RPE cells. Quantification of POS-positive vesicles in confocal z-stacks revealed vesicle diameters of 318 nm ± 69.2 for Rab-5, 421 nm ± 19.4 for Rab-7, 677 nm ± 32.1 for LAMP-1, 712 nm ± 59 for LAMP-2 and 990 nm ± 23.9 for LC3b [123]. The diameter of cargo-carrying vesicles in each compartment was consistent with the reported sizes of endosomes (200–500 nm) [15] and lysosomes (500 nm^–1^ μm) [29]. The increasing diameter of POS vesicles also indicated trafficking from early to more mature compartments, although this does not exclude the possibility of trafficking in Rab-5- and Rab-7-positive POS phagosomes. Indeed, the internalisation route of cargoes during early stages may largely be determined by the initial size of POS. For instance, it appears that POS may be trafficked through endosomes as smaller particles [125]. Hence, studies using POS assays necessitates a step to maximise single POS molecules or produce smaller POS particles prior to feeding cultures, as large POS aggregates remain bound to the apical RPE surface [125]. We also recoded the timing of POS trafficking through distinctive compartments in primary mouse RPE and ARPE-19 cells. Cargo internalisation and degradation in RPE cell-lines such as d407 and RPE-J are reportedly slower [135] compared to primary mouse/rat RPE [125,136]. Our timing experiments of POS internalisation and degradation in ARPE-19 cells also revealed slower kinetics compared to primary rodent RPE. Use of the pulse-chase assay enabled the initiation of POS internalisation to be synchronised following receptor binding. We observed the co-localisation of POS in Rab-5 compartments from 2 h with co-localisation in equal numbers within Rab-5- and Rab-7-positive vesicles by 6 h. POS in Rab-7 compartments diminished after 12 h whilst those in LAMP-1 and LAMP-2 vesicles appeared at 6–12 h and peaked between 12 and 24 h. POS co-localised to lysosomes characteristically accumulated in perinuclear regions (Figure 3E). POS eventually co-localised with LC3b-positive autophagy bodies after 12 h, which reached a peak at 48 h [123]. The development of novel imaging technologies as well as powerful new data-handling software provide the opportunity to reconstruct trafficking organelles in 3D (Figure 3). Others have also used a wide range of technologies to study cargo handling in the RPE [8,125,132,134]. Studies of this kind had posed considerable technical challenges in the past.

The end-products of POS degradation are absorbed by the RPE, recycled back to photoreceptors or removed from the cell. However, incomplete degradation of such waste material accumulates within RPE lysosomes as lipofuscin. Senescent post-mitotic macular RPE cells filled with lipofuscin are characteristic of the ageing retina and account for as much as ~20% of cell cytoplasmic volume by the eighth decade of life [137]. Lipofuscin disrupts the phagocytic mechanisms of RPE cells [138], impairs lysosomal proteases [139] and inhibits vATPase so that lysosomes become less acidic and cause leakage of contents into the cytosol [138]. Our work in neurons has revealed similar lysosomal damage leading to conditions such as AD [140]. Healthy macular RPE cells have higher levels of lysosomal enzymes acid phosphatase and cathepsin D relative to lysosomes from RPE in the nasal/mid-zone and peripheral retina [141]. However, the presence of lipofuscin causes lysosomal enzyme activity to decrease by up to 50% indicating the selective vulnerability of the macula to disease [142]. Lipofuscin also generates reactive oxygen species, which modify lipids and forms high molecular weight components that are stable within lysosomes [143,144,145]. Lipofuscin-containing compartments also contain molecules such as pyridinium bis-retinoid A2E [146,147], malondialdehyde (MDA) and 4-hydroxynonenal (HNE). A2E, a derivative of vitamin A [118,135], has the ability to irreversibly inhibit lysosomal cathepsin activity upon exposure to light [148]. MDA and HNE, by contrast, are generated as a result of lipid peroxidation of lipofuscins, and are capable of forming covalent bonds with adjacent proteins [149]. The accumulation of lipofuscin is thought to be partially caused by modification or crosslinking of proteins by MDA or HNE, thus reducing susceptibility to proteolysis [150]. Consequently, MDA and HNE modifications reduce POS clearance contributing to lipofuscin accumulation within lysosomes [151]. With respect to mechanisms through which protein aggregation may be dealt with, there appears to be no universal consensus as to whether autophagy plays a role, and whether its activity increases or declines with age and disease. The answer may lie in the cell-type, disease or indeed the specific stage of disease. Analysis of RPE or RPE/choroid in two AMD mouse models revealed an increase in autophagy markers LC3 I/II, SQSTM1/p62, ATG7, ATG9A as well as autophagosomes. Analysis of donor AMD tissues also showed an upregulation of LC3, ATG7 and ATG9 in the RPE [152]. Furthermore, drusen in AMD donor eyes contained markers of autophagy [9]. Interestingly, the actin motor protein myosin VI is expressed in the RPE layer and its deletion in a mouse model mimics an AMD-like phenotype, as characterised by an accumulation of basal-laminar deposits between the RPE and BrM [153]. Importantly, our earlier work revealed that myosin VI is required for autophagosome–lysosome fusion mediated by direct interactions with both autophagy and endocytic adaptor proteins [154]. This provides some corroborating evidence that targeted disruption of key autophagic regulators, for example the machinery essential for endosome and lysosome fusion, which results in autophagosome accumulation can lead to AMD-like disease phenotypes. Nonetheless, autophagy flux may increase or decrease depending on the capacity of RPE cells to cope with the elevated proteolytic stress in different stages of disease, which may further contribute to pathology. Readers are directed to several elegant reviews describing various mechanisms involved in these events [8,155]. 

## 6. Concluding Remarks 

New discoveries reveal lysosomes to be more than just end-points for cargo degradation. As discussed before, lysosomes have been shown to play an important role in nutrient sensing and cellular metabolism. Lysosomes also appear to have the ability to accumulate stores of cationic amino acids, polyphosphates, ions as well as other building blocks which can be released on demand [75]. The aforementioned multi-subunit mTORC1 complex is also capable of sensing amino acids both within and outside the organelle [156] and rapidly translocates to the lysosomal surface in response to amino acids and nutrients [157]. The discovery of a Zinc-finger transcriptional factor (ZKSCAN3) shed further light onto the complexity governing the aforementioned transcriptional regulation of lysosomes and the autophagosomes by nutrient-sensing molecules. ZKSCAN3 functions in an antagonistic manner to mTORC1 and translocates to the nucleus under nutrient-rich conditions to repress several lysosomal and autophagy genes [158]. Transcriptional regulators associated with lysosomes and autophagy thus play important roles in cellular energy balance [159] that may provide new insights into retinopathies. Investigators have also tested different approaches to manipulate lysosomal pH levels and bring about functional improvements in RPE cells [160], which could pave the way for future treatments. 

The sophistication of in-vitro RPE models have grown considerably such that they represent an excellent system to study how the endocytic and autophagy pathways become disrupted with disease. The RPE is also a good model for epithelial/barrier studies due to the availability of primary, stem cell-derived and transformed cell-lines which have been extensively characterized [161]. These cultures also enable a high degree of experimental manipulation and control, such that specific pathogenic conditions in the retina can be recapitulated for studies that would otherwise be difficult to undertake in whole mouse or donor eye tissues. Advances in creating adult-induced pluripotent stem cells such as those generated in our laboratory enable studies of RPE cells from patients [162], and the possibility of directly studying effects on the endocytic and lysosomal-autophagy pathways. Recent discoveries revealing new roles for lysosomes and autophagy are starting to provide insights into how factors such as energy metabolism and nutrition, that have hitherto garnered limited attention, could influence RPE cells in the senescent retina. These, coupled with advances in optics and novel imaging technology have created new possibilities in studying how cargo handling becomes impaired in RPE. Collectively, these have the potential to reveal fundamental new insights into a range of irreversible blinding conditions.
